# Selenium, Zinc, Copper, and Total Antioxidant Status in the Serum of Patients with Chronic Tonsillitis

**DOI:** 10.1007/s12011-016-0634-2

**Published:** 2016-02-05

**Authors:** Małgorzata Michalska–Mosiej, Katarzyna Socha, Jolanta Soroczyńska, Elżbieta Karpińska, Bogdan Łazarczyk, Maria Halina Borawska

**Affiliations:** 1 Department of Otolaryngology, Regional Hospital of Bialystok, M. Skłodowskiej-Curie 26 St., 15-950 Bialystok, Poland; 2 Department of Bromatology, Medical University of Bialystok, Mickiewicza 2D St., 15-222 Bialystok, Poland

**Keywords:** Chronic tonsillitis, Selenium, Zinc, Copper, Total antioxidant status

## Abstract

Antioxidants can play a significant role in chronic inflammatory process. The aim of this study was to evaluate the content of selenium (Se), zinc (Zn), copper (Cu), and total antioxidant status (TAS) of patients with chronic tonsillitis (CT). The study group consisted of 84 patients with CT from 18 to 62 years old and the control group of 67 healthy people aged 19–65 years. Se, Zn, and Cu concentration in serum samples were determined by atomic absorption spectrometry. Serum TAS was measured spectrophotometrically, using the test by Randox Laboratories-Us Ltd. The mean content of Se and Zn in the serum of patients with CT (61.122 ± 12.73 μg/L, 0.887 ± 0.26 mg/L, respectively) was lower compared to the control group (77.969 ± 12.73 μg/L, 0.993 ± 0.32 mg/L, respectively). The mean serum concentration of Cu in patients with CT (1.219 ± 0.35 mg/L) was higher compared to its serum concentration in healthy people (1.033 ± 0.37 mg/L). Serum TAS of patients with CT (1.171 ± 0.33 mmol/L) was lower in comparison with healthy volunteers (1.333 ± 0.42 mmol/L). The serum concentration of Se, Zn, and TAS in patients with CT was lower, whereas the concentration of Cu was higher compared to healthy volunteers. Smoking has an influence on reducing the concentration of Se and TAS of patients with CT.

## Introduction

Chronic tonsillitis (CT) is a frequently encountered disease which leads to throat pain, dysphagia, malaise, and fever [1]. The pathogenesis of CT is not exactly known, but during chronic inflammatory process, parameters of the immune system are impaired [2] with antioxidants playing a significant role in this process [3]. Low antioxidant levels may be the result of chronic diseases. The oxidation products are produced during inflammation and are involved in the tissue injury due to this inflammation. Free radical damage to the membrane lipids of leukocytes leads to its increased permeability and, therefore, decreases the immune function of leukocytes. Antioxidants play a role in neutralizing the destruction caused by these oxidation products [4]. Imbalance in concentrations of antioxidant elements in the human body is considered to be one of the risk factors of immune-related diseases.

Selenium (Se) is an essential trace element. Low Se intake and status have been shown to be associated with an elevated risk for various diseases. The key role of Se in human metabolism is attributed to its presence in glutathione peroxidase (GSH-Px)—an antioxidant enzyme.

The biological role of zinc (Zn) and copper (Cu) is related to their participation in the structures and functions of many enzymes, e.g., Cu/Zn superoxide dismutase with antioxidant and anti-inflammatory activity. Zinc affects multiple aspects of the immune system [5]. The ability of zinc to function as an antioxidant and stabilize membranes suggests its role in the prevention of free radical-induced injury during inflammatory processes [6]. During chronic Zn deficiency, the production of pro-inflammatory cytokines increases, influencing the onset of numerous inflammatory diseases [7].

Zn competes with Cu for binding to metallothionein, thereby reducing its absorption [8]. The role of Cu in the inflammation process has not been clearly defined. However, an inflammatory process control of the synthesis of free radicals is the most important in the interaction of Cu, resulting from the presence of this element in superoxide dismutase. In a chronic inflammatory process, increased phagocytosis enhances generation of free radical compounds, which leads to tissue damage polyunsaturated fatty acids, particularly susceptible to an attack of free radicals, are then oxidized. This leads to damage to biological membranes and to an increase in their permeability. Cu, reducing the release of lysosomal enzymes through the oxidation of thiols to disulfides membrane, causes a reduction in the permeability of biological membranes. Additionally, Cu affects the inflammatory process by binding to histamine, causing its reduced activity [9].

All antioxidants in the body (together enzymatic and non-enzymatic) form a total antioxidant status (TAS) of an individual. Miller et al. [10] defined total antioxidant status as the sum of endogenous and food-derived antioxidants of the extra cellular fluid of an individual. Cooperation of all the different antioxidants provides greater protection against the attack by reactive oxygen or nitrogen radicals than any single compound alone. The objective of this study is to evaluate the content of Se, Zn, Cu, and TAS of patients with CT.

## Material and Methods

The study was carried out in 84 patients with CT in the age of 18–62 years, qualified to the tonsillectomy and admitted to Otolaryngology Department, Regional Hospital of Bialystok. The control group consisted of blood samples from 67 healthy people aged 19–65 years. The patients and control group had no comorbid diseases such as diabetes, hypertension, and chronic obstructive pulmonary disease. The data of patients and controls are shown in Table 1.

**Table 1:** Patients and control group characteristic

Variable	Patients with CT (*n* = 84)	Control group (*n* = 67)
Gender (M/F)	30/54	23/44
Age (years)–mean (range)	36.23 ± 12.8 (18–62)	40.36 ± 14.0 (19–65)
No-smoking/smoking^a^	40/44	34/33

*M* male, *F* female

^a^Number of cigarettes, 5–20/daily; average duration of smoking, 15 years

Blood samples of patients were drawn before surgical procedure using the vacutainer system test tubes containing clot activator, Becton Dickinson, France. The samples were allowed to clot within 30 min then centrifuged within 10 min at approximately 1000×*g*. The serum was removed and kept frozen at −20 °C. The protocol of the study was approved by the Local Ethical Committee (R-I-002/575/2010).

The concentration of mineral components in the serum was determined by the electrothermal (Se, Cu) and flame (Zn) atomic absorption spectrometry method with Zeeman background correction (Z-2000 instrument, Hitachi, Japan). Certified reference material of human serum (Seronorm Trace Elements, Serum Level 1, 0903106, Sero AS, Norway) was used to test the accuracy of methods. The results of the quality control analyses corresponded with the reference values. The accuracy of the methods was 0.38, 0.94, and 0.55 and the coefficient of variation was 1.6, 2.4, and 1.2 % for Se, Cu, and Zn, respectively. The Department of Bromatology, Medical University of Bialystok participates in a quality control program for trace element analysis supervised by the National Institute of Public Health (Poland) and the Institute of Nuclear Chemistry and Physics (Poland). TAS in the serum was measured using the ready-made set of tests by Randox Laboratories-Us Ltd., USA and UV–Vis spectrophotometer (Cintra 3030, GBC, Australia). ABTS (2.2′-azino-di-[3-ethylbenzthiazoline sulphonate]) was incubated with a peroxidase (metmyoglobin) and H_2_O_2_ to produce the radical cation ABTS^+^. This had a relatively stable blue-green color, which was measured at 600 nm. Antioxidants in the added sample caused suppression of this color production to a degree which was proportional to their concentration [11].

Statistical analyses were performed using Statistica v.10.0 software. Differences between independent groups were tested by the Mann–Whitney *U* test. Values of *p* < 0.05 were considered significantly different. Correlation was calculated and tested by the Spearman rank test.

## Results

The mean content of Se and Zn in the serum of patients with CT (61.122 ± 12.73 μg/L, 0.887 ± 0.26 mg/L, respectively) was lower (*p* < 0.0000001, *p* < 0.03, respectively) compared to the control group (77.969 ± 12.73 μg/L, 0.993 ± 0.32 mg/L, respectively).

The average concentration of Cu in the serum of patients with CT (1.219 ± 0.35 mg/L) was higher (*p* < 0.002) in comparison with the serum of healthy people (1.033 ± 0.37 mg/L).

The TAS in serum of patients with CT (1.171 ± 0.33 mmol/L) was lower (*p* < 0.00004) compared to healthy volunteers (1.333 ± 0.42 mmol/L). A low correlation (*r* = 0.21, *p* < 0.05) was revealed between the concentration of Zn and TAS in the serum of the study group.

In the study, the lower concentration of Se and TAS (*p* < 0.02, *p* < 0.05, respectively) was established in the serum of smokers (57.876 ± 10.58 μg/L, 1.074 ± 0.28 mmol/L; respectively) compared to no-smoking patients (64.350 ± 14.44 μg/L, 1.244 ± 0.36 mmol/L; respectively) (Table 2).

**Table 2:** Content of Se, Cu, Zn, and TAS in the serum of patients with chronic tonsillitis (CT) and in the control group

No.	Subject	Number	Control group mean ± SD (A)	Number	CT patients mean ± SD (B)	*p* value ^A/B^ <
Selenium (μg/L)						
1.	All	67	77.969 ± 12.73	84	61.122 ± 12.73	0.0000001
2.	No-smoking	34	77.615 ± 17.82	40	64.350 ± 14.44	0.0000001
3.	Smoking	33	85.153 ± 24.53	44	57.876 ± 10.58*	0.0009
Copper (mg/L)						
4.	All	67	1.033 ± 0.37	84	1.219 ± 0.35	0.002
5.	No-smoking	34	1.006 ± 0.28	40	1.256 ± 0.32	ns
6.	Smoking	33	1.076 ± 0.46	44	1.193 ± 0.39	0.0006
Zinc (mg/L)						
7.	All	67	0.993 ± 0.32	84	0.887 ± 0.26	0.03
8.	No-smoking	34	0.888 ± 0.20	40	0.854 ± 0.28	ns
9.	Smoking	33	1.006 ± 0.38	44	0.924 ± 0.24	ns
TAS (mmol/L)						
10.	All	67	1.333 ± 0.42	84	1.171 ± 0.33	0.00004
11.	No-smoking	34	1.301 ± 0.40	40	1.244 ± 0.36	0.03
12.	Smoking	33	1.342 ± 0.34	44	1.074 ± 0.28^#^	0.02

*n* number of subject, *ns* not significant, *SD* standard deviation

*p_2/3_ < 0.02, ^#^ *p*_11/12_ < 0.05

In the study, a significant of correlation (*r* = 0.45, *p* < 0.0001) was proved between the content of Se in the serum and the age of patients with CT. Additionally, patients and the control group were divided into two age groups: under and over 40 years of age. In both groups, the concentration of Se was significantly lower compared to controls (*p* < 0.00003, *p* < 0.02, respectively). The concentration of Cu was higher in comparison with controls only in patients under 40 years old (*p* < 0.02), whereas TAS was significantly lower in patients with CT over 40 years compared to healthy volunteers (*p* < 0.01) (Table 3).

**Table 3:** Content of Se, Cu, Zn, and TAS in serum of patients with chronic tonsillitis (CT) and in the control group in two age groups—under and over 40 years of age

No.	Subject	Number	Control group mean ± SD (A)	Number	CT patients mean ± SD (B)	*p* value ^A/B^ <
Selenium (μg/L)						
1.	Under 40 years of age	33	75.005 ± 17.49	52	57.680 ± 10.90	0.000003
2.	Over 40 years of age	34	79.651 ± 22.23	32	66.706 ± 13.79	0.02
Copper (mg/L)						
3.	Under 40 years of age	33	1.031 ± 0.45	52	1.231 ± 0.37	0.02
4.	Over 40 years of age	34	1.064 ± 0.24	32	1.199 ± 0.33	ns
Zinc (mg/L)						
5.	Under 40 years of age	33	0.975 ± 0.33	52	0.906 ± 0.29	ns
6.	Over 40 years of age	34	0.933 ± 0.26	32	0.856 ± 0.21	ns
TAS (mmol/L)						
7.	Under 40 years of age	33	1.227 ± 0.39	52	1.163 ± 0.35	ns
8.	Over 40 years of age	34	1.405 ± 0.35	32	1.175 ± 0.31	0.01

*n* number of subject, *ns* no significant, *SD* standard deviation

## Discussion

The role of antioxidants in the pathogenesis of CT is not well understood. Free radical attack has been linked to numerous pathological conditions in all organs of the body, such as inflammation and infections. Reactive oxygen species are generated endogenously by inflammation and lipid peroxidation. Low antioxidant levels may predispose to the negative influence on the immune system. Se is an active component of various enzymes involved in redox reactions which protect membranes from oxidative damage. Deficit of Se may be connected with a risk of inflammatory diseases [12]. The reference level of Se in the serum is 70–140 μg/L [13]. In the study group, low Se concentration was observed in the serum, but in the control group, the average level of Se was within the reference range. The content of Se in a diet depends on the concentration of Se in the environment [14]. It has been well documented that Se levels in inhabitants from different regions of Poland are rather low [15]. Our earlier investigations showed that a degree of realization of the recommended daily allowance for Se (60 and 70 μg/day for women and men, respectively) was about half [16].

In the study group with CT, the level of Zn in the serum was lower, but the level of Cu was higher compared to the control group. Onerci et al. obtained similar results in children with chronic and recurrent tonsillitis [17]. The literature data relating to tonsillitis in children are available, but there are no data about adults. The reference level of Cu and Zn in the serum is 0.8–1.2 and 0.6–1.2 mg/L, respectively [13]. In patients with CT, the average concentration of Cu was above the reference levels, while the level of Zn was within the reference range. Conversely, serum concentration of TAS in patients with CT was below the references ranges for European population (1.30–1.77 mmol/L) [11]. Free radical attack has been linked to numerous pathological conditions in all organs of the body, such as inflammation and infections. Reactive oxygen species are generated endogenously by inflammation and lipid peroxidation. Low antioxidant levels may predispose to a negative influence on the immune system. There are a few studies on the role of antioxidants in CT in the available bibliography. Yilmaz et al. [4] estimated the levels of antioxidants (retinol, β-carotene, α-tocopherol, lycopene, ascorbic acid, superoxide dismutase, glutathione peroxidase, GSH) and peroxidation products (malondialdehyde) in children before and 1 month after tonsillectomy. Their results showed that the decreased preoperative blood levels of antioxidant vitamins and enzymes significantly increased postoperatively, while the increased preoperative blood level of oxidation product MDA significantly decreased postoperatively. The authors concluded that oxidants and antioxidants played a significant role in the pathogenesis of CT and the tonsillectomy significantly decreased the oxidative stress in patients. The studies suggest that the inflammatory process, through the production of reactive oxygen species, may deplete stores of antioxidants [18]. The issue whether increased consumption of foods rich in antioxidants or supplementation with antioxidants can provide health benefits requires further study. Polyphenols found in fruit and vegetables have a proven high antioxidant activity. In our study, patients who declared frequent consumption of vegetables and fruit had higher TAS compared to rare consumers of these products, but the difference was not statistically significant. Recent studies also indicate a positive role of the pro-oxidative properties of the flavonoids and thus their ability to form highly reactive quinones as an inductor of the expression of genes encoding detoxification enzymes [19, 20]. In our study, smoking cigarettes has a negative influence on the content of Se and TAS in patients with CT. Studies in Spain and Norway showed the lower level of Se in the serum among smokers [21, 22].

Further studies are necessary to estimate a possible therapeutic role of antioxidants, especially, dietary supplementation with Zn and Se, in preventing chronic tonsillitis.

## Conclusions

The concentration of Se, Zn, and TAS in the serum of patients with CT is lower, while the content of Cu is higher compared to healthy volunteers. Smoking has an impact on reducing the concentration of Se and TAS in the study patients.
